# Bacterial colonization of seromas after breast cancer surgery with and without local steroid prophylaxis

**DOI:** 10.1186/s12957-019-1661-1

**Published:** 2019-07-10

**Authors:** Christen Kirk Axelsson, Gro Maria Qvamme, Mette Okholm, Charlotte Lanng, Magnus Arpi, Minea Bruusgaard Mortensen, Birgitte Wegeberg, Pal Bela Szecsi

**Affiliations:** 1 Department of Breast Surgery F, University of Copenhagen, Herlev Hospital, Herlev, Denmark; 2 Department of Clinical Microbiology, University of Copenhagen, Herlev Hospital, Herlev, Denmark; 3 Department of Clinical Biochemistry, University of Copenhagen, Holbæk Hospital, Holbæk, Denmark

**Keywords:** Seroma, Breast cancer, Breast neoplasm, Surgery, Surgical wound infection, Microbiology, Steroid

## Abstract

**Background:**

Seroma formation is a frequent postoperative sequela after mastectomy for primary breast cancer. We investigated the role of bacterial colonization of seroma fluid with three different culture methods and the effect of intracavitary steroids.

**Methods:**

The study group consisted of 212 patients scheduled for mastectomy from a previously performed double-blind randomized placebo-controlled intervention trial. The patients were allocated to a single dose of 80 mg of steroids (methylprednisolone) or saline, and the effect on seroma formation was investigated. From each aspiration, an equal volume of seroma fluid (10 mL) was distributed into one sterile transport tube (conventional method), one aerobic blood culture bottle and one anaerobic blood culture bottle.

**Results:**

There was significant variation in the number of bacterial species detected in seroma samples among the three culture methods, ranging from 18 species with the conventional culture tubes to 40 species with aerobic blood culture bottles. Patients receiving prophylactic steroids had significantly more frequent colonization than those in the saline group. Nevertheless, the clinical surgical site infection rate of 7.0% was equal between the two groups.

**Conclusions:**

In general, data analysis of the entire set of case material did not succeed in demonstrating a relationship between a specific bacterial species or a combination of species and seroma formation. However, in the few patients with growth of a pathogenic species, both the duration of seroma formation and volume of seroma fluid were more pronounced.

Trial registration: Ethics Committee of Copenhagen (H-4-2009-137), (EudraCT number 2009-016650-40), the Danish Data Protection Agency (code J. no. F.750.75-2), and the Danish Health and Medicines Authority (sponsor protocol code number 23837). Start date November 2010.

## Background

Seroma formation is the most prevalent complication after mastectomy and axillary lymph node dissection and is often regarded as an unavoidable nuisance rather than a serious complication [1, 2]. However, seromas result in patient discomfort and repeated hospital visits and may delay adjuvant therapy.

Seroma formation has an acute inflammatory component in response to surgical trauma [3, 4] via the activities of proteinases, proteinase inhibitors, cytokines, and growth factors [5, 6]. In the early postoperative period, seroma fluid slowly becomes exudative with some characteristics similar to those of lymph [7]. Decreasing the inflammatory response with steroids has been successfully performed for major abdominal surgery [8], colonic resection [9], head and neck surgery [10], plastic surgery [11], and cardiac surgery [12]. Local administration of the steroid triamcinolone into the wound cavity after autologous *latissimus dorsi* breast reconstruction has been shown to reduce seroma formation [13], whereas a single intravenous bolus of steroids (methylprednisolone) before mastectomy exerted no effect [14]. Steroid administration into the wound cavity on the first day after mastectomy with sentinel lymph node biopsy exerted a significant preventive effect against seroma formation during the next 30 days, while no effect was observed after mastectomy with axillary lymph node dissection [15].

This study evaluates the hypothesis that subclinical bacterial colonization is a contributing pathogenic factor to seroma formation after mastectomy with axillary lymph node dissection and evaluates whether steroid prophylaxis increases the risk of subclinical infection. Furthermore, we investigated whether repeated seroma punctures introduce opportunistic subclinical infection, contributing to persistence of seromas.

## Materials and methods

### Study group

This observational study was a part of a double-blind randomized placebo-controlled intervention study described in detail previously [15, 16]. Briefly, the original study (EudraCT number 2009-016650-40) analyzed the effect on seroma formation after mastectomy of a single dose of 80 mg of steroids into the mastectomy cavity. A total of 212 patients were randomized. The per-protocol study group consisted of 100 patients in the saline group and 99 patients in the steroid group. The study demonstrated that injection of methylprednisolone acetate on the first day after mastectomy and sentinel node biopsy had a significant effect on the prevention of seroma formation. Steroid administration more than halved the number of patients who did develop a seroma, reduced the number of aspirations required, and decreased the mean and cumulative seroma volume within both 10 and 30 days. The effect of steroid was not seen in the mastectomy and axillary level 1–2 dissection group.

### Microbiological examination

Seroma puncture was performed in a sterile closed system. One aerobic and one anaerobic blood culture bottle (BACTEC^TM^, Becton Dickinson, Franklin Lakes, NJ, USA) were each inoculated with 10 mL of seroma fluid immediately after collection and an additional 10 mL were collected in a sterile transport tube (conventional method). The samples were transported to the Department of Clinical Microbiology as fast as possible, and kept at room temperature (bottles) or at 4 °C (transport tube) until arrival. At arrival, the seroma fluid in the transport tube was cultured on a 5% blood agar plate and a chocolate plate (Oxoid, Basingstoke, Hampshire, UK). Plates were examined after incubation for 1 and 2 days at 35 °C and 5% CO_2_. Aerobic and anaerobic blood culture bottles were incubated for 5 days in the BACTEC^TM^ instrument. When the instrument indicated positivity, the bottle was subcultured on the same plates as the specimen in the transport tube. All isolates were identified to the species level with matrix-assisted laser desorption/ionization–time of flight (MALDI-TOF; Bruker Daltonics, Billerica, MA, USA).

*Staphylococcus aureus*, β-hemolytic streptococci, *Enterobacteriaceae*, and *Pseudomonas aeruginosa* were considered likely pathogenic bacteria. Common skin commensal bacteria (e.g., coagulase-negative staphylococci (except for *Staphylococcus lugdunensis*), *Micrococcus* species, *Corynebacterium* species, *Propionibacterium* species) were regarded as contaminants, except when the same species was isolated in two time-separated cultures within a 5-day period [17]. Quantitative culture of seroma fluid from the transport tube was not performed. The culture results from the research specimens were concealed from the clinicians until the end of the study unless a clinical surgical site infection (SSI) occurred and in a single patient with growth of *P. aeruginosa.* The use of antibiotics was solely a clinical decision based on the presence of a combination of fever, redness of the wound, and/or purulent seroma fluid.

### Statistics

Groups were compared using one-way analysis of variance (ANOVA). Levene’s test for homogeneity of variance, and post hoc analyses with Tamhane’s T2, assuming unequal variances and group sizes, were used to investigate the nature of any differences. Associations of categorical factors were analyzed using the chi-square test or Fisher’s exact test and calculation of odds ratios (ORs) and 95% confidence intervals (CIs). A two-tailed value of *p* < 0.05 was considered statistically significant. Data analyses were performed using SPSS software Version 22.0 (IBM, Armonk, NY, USA).

## Results

### Study demography

During the study period, 881 seroma aspirations (439 in the saline group and 442 in the steroid treatment group) were evaluated by microbial culture. These aspirations were performed in 141 patients (82 patients in the saline group and 59 in the steroid treatment group). On average, each of these patients had a seroma aspirated 8 times (range, 1–51 times), including 8.4 times in the saline and 7.6 times in the steroid group. The findings during the entire study period from the examination of 2643 seroma cultures distributed among the three culture methods are presented in Table 1.

**Table 1:** Microbiologic findings in all 881 seroma samples after primary breast cancer surgery in 199 patients expressed as a percentage of positive and negative cultures in the groups treated with steroids (methylprednisolone) or saline. Microorganisms in seroma cultures were identified with a conventional method and with aerobic and anaerobic blood culture bottles. The results of each of the culture methods (counts and percentages) are shown in relation to the combined evaluation of all three methods and classified according to the microbiological finding and treatment group. Data were not available in some cases for all three microbiological methods. The present study was part of a double-blind randomized placebo-controlled intervention study that analyzed the effect of a single dose of 80 mg of steroids on seroma formation after mastectomy

	Culture method					
	Conventional method^a^		Aerobic blood culture bottle^b^		Anaerobic blood culture bottle^c^	
Treatment	Neg *n* = 746 *n* (%)	Pos *n* = 131 *n* (%)	Neg *n* = 645 *n* (%)	Pos *n* = 230 *n* (%)	Neg *n* = 656 *n* (%)	Pos *n* = 214 *n* (%)
Saline	406 (46.1)	31 (3.5)	363 (41.2)	73 (8.3)	368 (41.8)	65 (7.4)
Steroids	340 (38.6)	100 (11.4)*	282 (32.0)	157 (17.8)*	288 (32.7)	149 (16.9)*
Saline vs steroids odds ratio (95% CI)	6.58 (3.82–11.34)		5.02 (3.30–7.62)		4.74 (3.11–7.24)	

^a^Data not available in 4 cases (0.5%)

^b^Data not available in 6 cases (0.7%)

^c^Data not available in 11 cases (1.2%)

*Positive culture steroids vs saline, Fisher’s exact test *p* < 0.001

### Surgical si te infection (SSI)

Seven patients in both the steroid and saline groups were treated with antibiotics due to a surgical site infection SSI during the first 30 days postoperatively. The median time of occurrence of a SSI was on the 15th day (range, 3–23 days) postoperatively in the saline group and on the 17th day (range, 11–24 days) postoperatively in the steroid group. SSIs occurred after more than 30 days postoperatively in six patients in the saline group after 8–36 aspirations after 33–110 days postoperatively. In the steroid group, SSIs occurred in 11 patients after 5–24 aspirations after 41–153 days postoperatively. Among these patients, two patients had reinfections, including one patient in the saline group at 70 days postoperatively and one patient in the steroid group at 85 days postoperatively. All patients recovered following antibiotic treatment. No differences in the frequency of SSIs between the saline and steroid groups before or after 30 days postoperatively were observed.

### Culture methods

The findings by the three culture methods are presented in Table 1. Data were not available in four cases (0.5%) from the conventional culture method, in 6 cases (0.7%) from aerobic blood culture bottles and in 11 cases (1.2%) from anaerobic blood culture bottles. The conventional culture method resulted in a significantly lower detection rate than both aerobic blood culture bottles and anaerobic blood culture bottles (Fisher's exact test *p* < 0.0001) (data not shown). No significant differences in the detection rate between aerobic and anaerobic blood culture bottles were observed.

### Effect of steroid

Steroid treatment resulted in significantly more positive seroma cultures than saline as assessed by the conventional method (*p* < 0.0001, OR 6.58, 95% CI 3.82–11.34), aerobic blood culture bottles (*p* < 0.0001, OR 5.02, 95% CI 3.30–7.62), and anaerobic blood culture bottles (*p* < 0.0001, OR 4.74, 95% CI 3.11–7.24) (Table 1). Among the 532 seroma aspirations (300 in the saline group and 232 in the steroid group), that occurred within the first 30 days after surgery, 63 aspirations (11.8%) and 118 aspirations (22.2%) in the saline and steroid groups, respectively, were positive with at least one of the three culture methods. Positive cultures occurred significantly more frequently in the steroid treatment group (Fisher's exact test *p* < 0.0001, OR 3.90, 95% CI 2.66–5.69).

### Microbiological colonization

The spectrum of bacterial species varied among the three culture methods (Table 2). The number of bacterial species in monomicrobial and polymicrobial cultures ranged from a minimum of 18 (conventional culture) to 32 (anaerobic blood culture bottle) to a maximum of 40 species (aerobic blood culture bottle), among which *S. epidermidis* was found in nearly half of all positive cultures. There was a similar species distribution in the steroid and saline groups, but there was a higher frequency in the steroid group (*p* < 0.0001); *S. epidermidis* was again the dominant species identified in nearly half of the positive cultures. Employing aerobic and anaerobic blood culture bottles increased the identification rates of staphylococci and streptococci with a factor of two to three. *S. aureus* was the next most frequently identified pathogen, and both species were observed three times more frequently in the steroid group than in the saline group.

**Table 2:** Species distribution of microorganisms in seroma cultures performed with a conventional method and with aerobic and anaerobic blood culture bottles. Microbiologic findings in 881 seroma samples after primary breast cancer surgery in 199 patients. The present study was part of a double-blind randomized placebo-controlled intervention study that analyzed the effect of a single dose of 80 mg of steroids on seroma formation after mastectomy

Organism	Conventional method		Aerobic blood culture bottle		Anaerobic blood culture bottle	
Treatment	Saline	Steroids	Saline	Steroids	Saline	Steroids
No growth (*n*)	408	340	363	283	368	288
Not performed (*n*)	1	3	2	4	3	2
Positive culture (*n*)	30	100	74	156	66	149
Isolates (*n*)	31	102	78	162	69	154
*Staphylococcus aureus*	5	17	6	19	6	21
Coagulase-negative staphylococci - *Staphylococcus epidermidis* - *Staphylococcus lugdunensis*	18 13 1	74 48 1	51 29 4	115 68 3	46 31 2	113 70 1
*Micrococcus* spp.	0	0	4	1	0	0
*Rothia* spp.	0	0	1	1	1	0
β-hemolytic streptococci	2	3	2	3	2	2
Non-hemolytic streptococci	0	2	4	10	7	9
*Corynebacterium* spp.	0	1	1	7	0	0
*Bacillus* spp.	0	2	3	2	1	1
*Lactobacillus* sp.	0	0	0	0	0	1
*Enterobacter cloacae*	0	2	0	1	0	3
*Pseudomonas* spp.	1	0	4	2	4	1
*Acinetobacter* sp.	0	0	1	0	0	0
Anaerobes	4	1	1	1	2	3

### Colonization over time

**Fig. 1:**
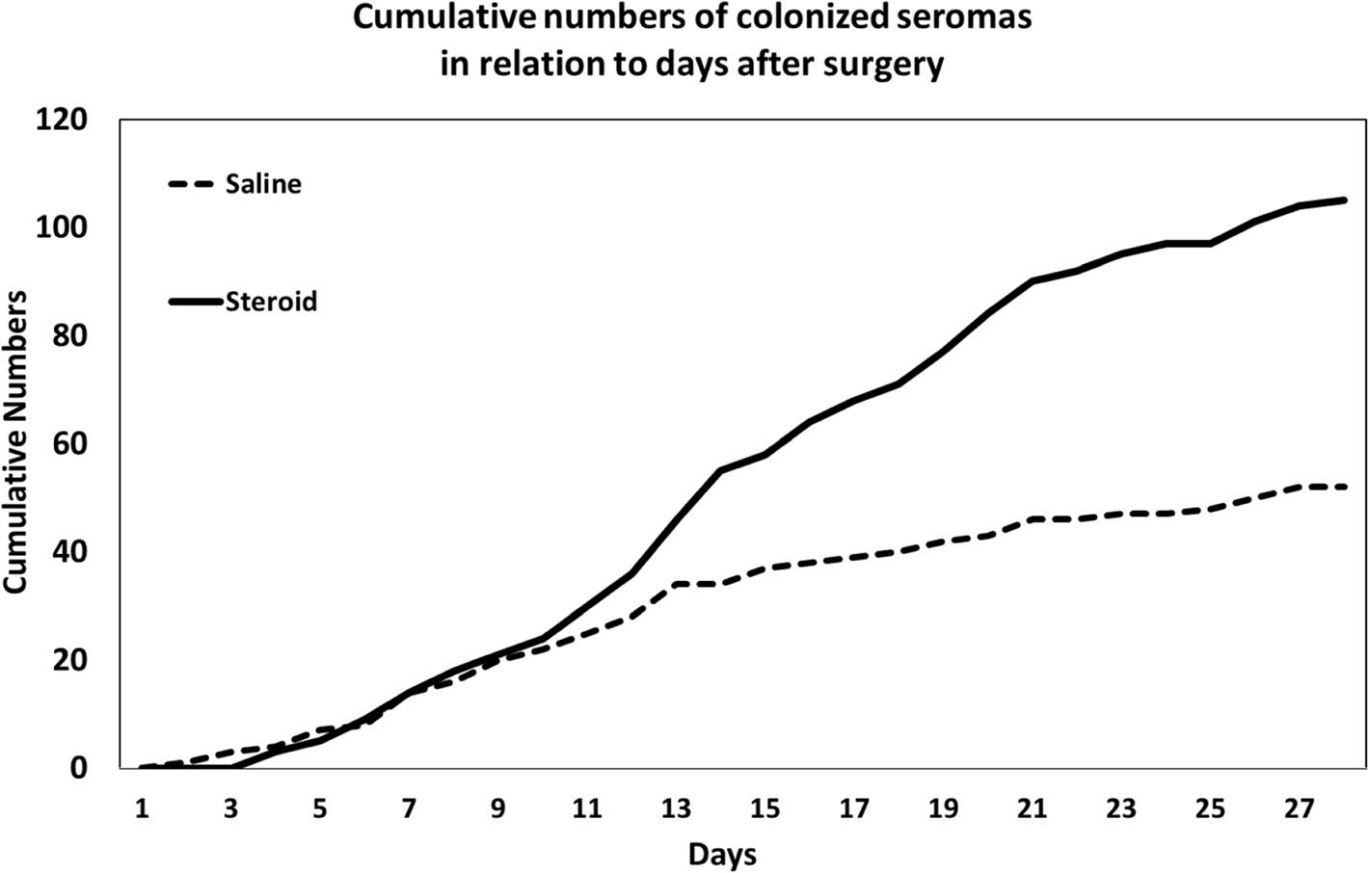
Cumulative numbers of colonized seromas among patients in the saline group and the steroid group identified with at least one of the three culture methods including the conventional method and aerobic and anaerobic blood culture bottles during the first 30 days postoperatively in relation to the number of days after surgery. The difference between the two treatment groups was significant, *p* = 0.002. The present study was part of a double-blind randomized placebo-controlled intervention study that analyzed the effect of a single dose of 80 mg of steroids on seroma formation after mastectomy

The rate of positive cultures was constant in both treatment groups up until approximately the tenth day after surgery. After this time, a decrease in the positive culture rate occurred in the saline group, while the initial rate continued to increase in the steroid group until approximately the twenty-fifth day, (ANOVA‚ steroid vs. saline, *p* = 0.002, Fig. 1). Similarly, the cumulative aspirated volume of seromas with positive cultures was equal in both groups until the twelfth day. After this time, a decrease in cumulative volume occurred in the saline group, while the initial rate continued to increase in the steroid group.

A group of 48 patients with positive cultures and without SSIs or the need for antibiotic treatment underwent aspiration 99 times. They were followed for a median of 16 days (range, 2–57 days) with a median of three aspirations (range, 1–9 positive aspirations) before seroma formation ceased. *S. epidermidis* was identified in 19 patients, other coagulase-negative staphylococci were identified in 14 patients, and *Corynebacterium* species were identified in five patients. In the remaining ten patients, miscellaneous species were identified.

All culture results were classified according to probable clinical significance, i.e., contaminant microorganism vs. potentially pathogenic microorganism. A significant difference between the two treatment groups of patients was demonstrated. In the saline group, a higher frequency of contaminant isolates was found. Conversely, in the steroid group, a higher frequency of potentially pathogenic isolates was found (*p* < 0.001, Table 3). No significant differences in the seroma duration, total cumulative seroma volume, or cumulative seroma volume in the first 10 or 30 days after surgery were observed (*p* = 0.5) between women with negative cultures and those with any positive cultures (pathogenic or contaminant isolates). However, compared to women with negative cultures (*n* = 125), women with growth of pathogenic bacteria (*n* = 59) had a significantly longer seroma duration (42.7 days, *p* = 0.013), larger total cumulative seroma volume (2255 mL, *p* = 0.004), and a larger cumulative seroma volume in the first 10 days (185 mL, *p* = 0.004) or 30 days (983 mL, *p* = 0.000) after surgery.

**Table 3:** Microorganisms in seroma cultures performed with a conventional method and with aerobic and anaerobic blood culture bottles. Each positive seroma culture was classified according to the microbiological finding as “most likely irrelevant” (no growth or contamination) or as “most likely pathogenic” (potentially pathogenic or pathogenic). The present study was part of a double-blind randomized placebo-controlled intervention study that analyzed the effect of a single dose of 80 mg of steroids on seroma formation after mastectomy

	Culture results	
	Bacterial classification	
Treatment	Contaminant microorganism *n* (%)	Most likely pathogenic microorganism *n* (%)
Saline	254 (49.0%)	39 (7.5%)
Steroids	213 (5.1%)	95 (18.3%)*
Saline vs steroids odds ratio (95% CI)	4.76 (3.10–7.31)	

*Significant more “probably pathogenic” growth occurred in the steroid group (Fisher’s exact test *p* < 0.001).

## Discussion

Through the decades, seroma formation after breast surgery has been a topic of discussion. Most papers have focused on how to avoid or how to treat seromas [18, 19]. The microbiological spectrum and subclinical bacterial colonization as risk factors for seroma formation have not gained much attention. The present study is one of few studies to present a systematic and longitudinal study of the microbiological spectrum present in seromas after breast surgery [20]. Three different culture methods were investigated, and a variation in species was detected. The aerobic and anaerobic blood culture methods revealed a wide spectrum of species compared with the conventional culture method (Table 2). The increased number of different species observed with both aerobic and anaerobic blood culture bottles is of scientific interest, but this finding goes beyond daily clinical routines. The finding that the overall frequency of negative cultures evenly declined from the first puncture to the approximately 45th puncture in individual patients is in accordance with the theory that seromas are associated with a slowly declining inflammatory reaction. The finding that the rate of positive cultures was nearly constant during the first 50 days indicates that perioperative subclinical colonization occurs despite aseptic technique. However, during the first 30 days after surgery, the rate of seroma aspirations with microbial growth was noticeably lower after the 12th day postoperatively in the saline group than in the steroid group (Fig. 1). Similarly, the seroma volume decreased in the saline group after the 10th day. This finding indicates that steroid treatment has some adverse effects on seroma formation among the patients with positive cultures, even though this effect was not observed during the first 10–12 days or in the overall population. However, the follow-up of the group of 48 patients with positive seroma cultures but normal clinical findings and the presence of potential pathogenic bacteria showed that seroma formation decreased spontaneously. This result indicates that culture-positive findings alone are not an automatic indication for antibiotic treatment. The dominant microbiological species identified in the present study were *S. epidermidis* and *S. aureus*. While nosocomial infections with *S. epidermidis* have gained much attention, it has been suggested that this skin colonizer has apparently not evolved to cause disease but maintains a benign relationship with its host [21]. *S. epidermidis* is generally associated with catheters and other medical implants. With such characteristics, *S. epidermidis* could be hypothesized to be a possible provoking factor for seroma formation. This study did not confirm this hypothesis, and these bacteria were only involved in two cases of SSIs.

The association between women with negative cultures compared to positive cultures with pathogenic isolates could support a causal factor on seroma production. However, the finding is based on small numbers of cultivations so conclusions cannot be drawn, and furthermore it is likely that the more frequent and longer seroma formation has a higher risk for having pathogenic bacteria introduced.

In planning the study, the use of steroids was hypothesized to be a risk factor for infection, and our results confirmed that colonization of seromas in the steroid group was more frequent. Frequent colonization was also observed in the saline group, but to a lesser degree. However, notably, only 14 of 199 patients (7.0%) developed SSIs within the first 30 days after surgery (the maximal duration of steroid action), with no overrepresentation in the steroid group. The patients were only treated with antibiotics on a clinical basis. The incidence of SSIs in breast cancer surgery is reported to range between 3% [22] and 15% [23, 24]. The present finding of a 7% incidence of SSIs is higher than the reported 3.4% rate associated with clean surgical techniques [25]. However, the use of prophylactic antibiotics to prevent infection is still a controversial issue, and routine use is not common in breast cancer surgery. Suboptimal prophylactic antibiotics may increase the risk of infection [26]. A large series of reduction mammoplasty recommended a single intravenous perioperative dose of antibiotics with action against *S. aureus* [24]. In 2014, a Cochrane review of 11 studies (2867 participants) demonstrated that prophylactic antibiotics administered preoperatively significantly reduced the incidence of SSIs among patients undergoing breast cancer surgery without reconstruction (pooled risk ratio (RR) 0.67, 95% CI 0.53–0.85) [23]. A short Cochrane review of a single study comparing perioperative antibiotics with no antibiotics found no statistically significant effect of antibiotics on the incidence of SSIs (RR 0.11, 95% CI 0.01–1.95) [27]. A recent Australian retrospective cross-sectional study reported a growing adherence to surgical antibiotic prophylaxis guidelines in breast surgery and concluded that the incidence of SSIs was low, and there was no relationship with adherence to the guidelines [28], indicating that prophylaxis was effective [14]. In contrast, the present study indicates that antibiotic treatment, based solely on clinical observations, was effective and safe. Had our study been based on culture results, we estimate that two or three as many patients would have received redundant antibiotic treatment. Our results from present and earlier studies [14–16] combined with other results [13] led to recommendations for seroma prophylaxis with intracavitary steroids with optional repeated steroid administration if the seroma persists.

## Conclusions

In summary, our data analysis on the case material did not succeed in demonstrating a relationship between a specific bacterial species or combination of species and seroma formation. Instillation of steroids on the first postoperative day resulted in frequent colonization with various species compared with instillation of saline. These findings do not support any routine culture schedule for every seroma sample or the necessity of prophylactic antibiotic treatment due to seroma prophylaxis with steroids. Empirical clinical evaluation of the indications for antibiotic treatment for infected seromas was sufficient and safe.

## Data Availability

Datasets from the current study are available from the corresponding author on reasonable request.

## Abbreviations

ANOVA: Analysis of variance
CI: Confidence interval
MALDI-TOF: Matrix-assisted laser desorption/ionization—time of flight
OR: Odds ratio
RR: Pooled risk ratio
SSI: Surgical site infection
